# The distribution of mitochondrial DNA haplogroup H in southern Iberia indicates ancient human genetic exchanges along the western edge of the Mediterranean

**DOI:** 10.1186/s12863-017-0514-6

**Published:** 2017-05-19

**Authors:** Candela L. Hernández, Jean M. Dugoujon, Andrea Novelletto, Juan N. Rodríguez, Pedro Cuesta, Rosario Calderón

**Affiliations:** 1 Departamento de Zoología y Antropología Física, Facultad de Biología, Universidad Complutense, Madrid, Spain; 2 CNRS UMR 5288 Laboratoire d’Anthropologie Moléculaire et d’Imagerie de Synthèse (AMIS), Université Paul Sabatier Toulouse III, Toulouse, France; 3 Dipartimento di Biologia, Università Tor Vergata, Rome, Italy; 4 Servicio de Hematología, Hospital Juan Ramón Jiménez, Huelva, Spain; 5 Centro de Proceso de Datos, Universidad Complutense, Madrid, Spain

**Keywords:** Gene flow, Phylogeography, Population structure, Iberian Peninsula, North Africa, Human evolution

## Abstract

**Background:**

The structure of haplogroup H reveals significant differences between the western and eastern edges of the Mediterranean, as well as between the northern and southern regions. Human populations along the westernmost Mediterranean coasts, which were settled by individuals from two continents separated by a relatively narrow body of water, show the highest frequencies of mitochondrial haplogroup H. These characteristics permit the analysis of ancient migrations between both shores, which may have occurred via primitive sea crafts and early seafaring. We collected a sample of 750 autochthonous people from the southern Iberian Peninsula (Andalusians from Huelva and Granada provinces). We performed a high-resolution analysis of haplogroup H by control region sequencing and coding SNP screening of the 337 individuals harboring this maternal marker. Our results were compared with those of a wide panel of populations, including individuals from Iberia, the Maghreb, and other regions around the Mediterranean, collected from the literature.

**Results:**

Both Andalusian subpopulations showed a typical western European profile for the internal composition of clade H, but eastern Andalusians from Granada also revealed interesting traces from the eastern Mediterranean. The basal nodes of the most frequent H sub-haplogroups, H1 and H3, harbored many individuals of Iberian and Maghrebian origins. Derived haplotypes were found in both regions; haplotypes were shared far more frequently between Andalusia and Morocco than between Andalusia and the rest of the Maghreb. These and previous results indicate intense, ancient and sustained contact among populations on both sides of the Mediterranean.

**Conclusions:**

Our genetic data on mtDNA diversity, combined with corresponding archaeological similarities, provide support for arguments favoring prehistoric bonds with a genetic legacy traceable in extant populations. Furthermore, the results presented here indicate that the Strait of Gibraltar and the adjacent Alboran Sea, which have often been assumed to be an insurmountable geographic barrier in prehistory, served as a frequently traveled route between continents.

**Electronic supplementary material:**

The online version of this article (doi:10.1186/s12863-017-0514-6) contains supplementary material, which is available to authorized users.

## Background

The particular spatial distribution patterns of specific mitochondrial lineages in contemporary human populations offer insights into human origins, past migration events and gene flow with defined directions and demographic consequences [1–5]. Mitochondrial macro-haplogroup H (Hg H) has been a focus of attention in human genetic diversity studies for more than a decade [6–9]. Examining the spatial distribution of H lineages and other features associated with its evolutionary history have been pivotal in understanding the formation of the western European gene pool. Early works revealed many features of this genetic cluster, which comprises a star-like phylogeny composed of a central, major node with many rarer variants that arise from it [10, 11]. The estimated coalescence time for Hg H (~21,000 years ago, ya) has led to the proposal that the clade was involved in a post-glacial population re-expansion from southwestern Europe to the rest of the continent.

Hg H (the native *Euroasiatic* marker par excellence) clearly dominates the mitochondrial DNA (mtDNA) gene pool of Europeans (~40-45% on average) [8, 9]. Hg H has an internally complex structure, with regional geographic specificities across Europe and the Mediterranean Basin. The patterns of variation revealed by H lineages (and sub-lineages) were better characterized as more refined molecular technologies were developed, which enabled, for example, screening of coding region Single Nucleotide Polymorphisms (SNPs) [7, 8] and complete sequencing [6]. This technical progress increased phylogenetic resolution, thus demonstrating that *i*) the number of internal branches shaping clade H is significantly greater than in other mtDNA Hgs widespread in Europe [7], and *ii*) the observed Hg H variation in eastern regions (e.g., Near/Middle East and Caucasus) shows marked differences to that found in western Europe [9]. Moreover, the classification of H mtDNA samples in sub-lineages with only control region variants has proven in most cases to be unreliable due to the recurrence of some polymorphisms and the absence of diagnostic sites [7].

Basques and other neighboring populations from the northern Iberian Peninsula have been excellent candidates for studying Hg H composition in western Europe [12–14]. These populations are presumed to be a source of the European post-glacial peopling signaled by some H sub-Hgs. For example, frequency peaks of mtDNA lineages H1 and H3 characterize Cantabrian/Iberian populations. In this line, Basques are presumably the native population of derived sub-branches emerging from H1 and H3, including H1j1, H1t1 and H3c2a. These, together with H2a5, compose ~40% of Hg H and are absent in other populations [13].

Hg H lineages are also dispersed outside of Europe. A pertinent example is found in North Africa, where Hg H is the main marker of the European influence across the Maghreb [15, 16]. Most North African H sequences belong to sub-Hgs H1 (42%) and H3 (13%) [17]; thus Hg H seems to be structured here in much the same way as in Iberia. Nevertheless, some surveys [16] have shown that great genetic diversity could be hidden in Hg H profiles among northern Africans. It has also been assumed that a portion of H lineages in the region were transferred by a post-glacial wave of expansion from the Franco-Cantabrian/Iberian region southward. Thus, a detailed analysis of this clade in populations located along this supposed migration route is crucial. We present here for the first time a comprehensive, high-resolution phylogenetic portrait of Hg H in Andalusia; this region would have been midway between the area of departure and the southernmost limit of mitochondrial H sequences in North Africa. It is thus possible to explore whether the southernmost region of Spain served as a stopover for Hg H descendant lineages in their intercontinental route or if other maternal genes underwent direct, non-mediated migration to North Africa, either through the Strait of Gibraltar or the surrounding maritime region. For this analysis, we selected two autochthonous populations from Huelva and Granada provinces, which are geographically placed at the western and eastern ends of Andalusia, respectively. The global mtDNA variation in the two territories exhibits significant genetic differentiation [18]. The population structuring seems to be primarily caused by the differential weight of African lineages U6, M1 and L, which are far more represented in the western than in eastern Andalusia. The complete sequencing of African mtDNA lineages found in the Andalusian gene pool [19] have interestingly revealed the occurrence of ancient trans-continental contact between northwestern Africa and Iberia, with Andalusia being the Atlantic side of the Peninsula where most African maternal traces are concentrated when compared to the rest of Europe.

This study attempts to provide an accurate picture of the distinctiveness of the Andalusian matrilineal gene pool in comparison to other Iberian and Mediterranean populations. The essential role played by Iberia in disseminating specific lineages into North Africa through migrations since prehistoric times is supported here by mtDNA Hg H.

## Methods

### Populations and sample selection

**Fig. 1:**
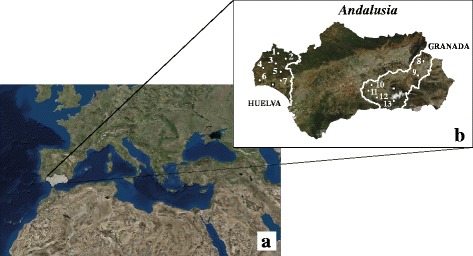
Map of the region. **a** Location of the Andalusian territory in the context of the Mediterranean region. **b** Municipalities of sampling in Huelva and Granada provinces: 1: El Repilado, 2: Aracena, 3: El Cerro del Andévalo, 4: La Puebla de Guzmán, 5: Valverde del Camino, 6: Villablanca, 7: Niebla, 8: Huéscar, 9: Baza, 10: Montefrío, 11: Loja, 12: Alhama de Granada, 13: Órgiva. The capital cities are highlighted with a square. Basemaps were adapted from ArcGIS Online repository (World Imagery, Esri) and NASA Visible Earth (Spain and Portugal. Credit: Jacques Descloitres, MODIS Land Rapid Response Team, NASA/GSFC. https://visibleearth.nasa.gov/). Both images are freely available for re-publication or re-use

Blood samples (*n* = 750) were collected between 2004 and 2009 in the provinces of Huelva (10,147 km^2^) and Granada (12,635 km^2^), located in western and eastern Andalusia, respectively (see Fig. 1). Volunteer donors were recruited by doctors and nurses from the Juan Ramón Jiménez Hospital in Huelva and the Provincial Blood Transfusion Center in Granada, with the assistance of researchers from Complutense University of Madrid. The Bioethics Committee of the Complutense University of Madrid has approved the research protocols used for this study. All subjects (donors) provided signed informed consent for sample collection. Each donor was informed of the main scientific goals of the study and was kindly asked to indicate the geographic origin of his/her family. Subjects screened in the present study were healthy, unrelated and autochthonous (maternal ancestry recorded for a minimum of three generations). Parents and grandparents of the donors were born in 48 of 79 and 57 of 169 different municipalities from the Huelva and Granada provinces, respectively. Figure 1 depicts the localities represented in the overall sample.

### Molecular characterization of Hg H and its sub-clades

Out of 750 subjects, 337 (110 from Huelva and 227 from Granada) belonged to mtDNA Hg H. Two TaqMan SNP genotyping assays (Life Technologies, Carlsbad, CA, USA) were designed to genotype T7028C (Hg H) and G3010A (sub-Hg H1) polymorphisms. Conditions for reactions, as well as primers and probes, are reported in Additional file 1. We sequenced the mtDNA hypervariable region I (HVS-I) and part of HVS-II (pos. 16023-273), following conditions described in [20]. A subset of samples (*n* = 113) came from a previous study [18]. The 224 new control region sequences have been deposited in the GenBank database (Accession numbers KY992104 - KY992327).

Here, 21 SNPs within Hg H were screened: G750A, G951A, G3915A, C3992T, T4336C, G4769A, A4793G, T6365C, T6776C, A8271t, T8473C, T8602C, A9066G, A9150G, C12858T, T12957C, G13708A, G13759A, C14365T, C14872T, A13101c (see the sub-Hgs tested in Additional file 2). We specifically selected those polymorphisms that define the most common H sub-branches in southwestern Europe. The molecular variants were genotyped using the SNaPshot Multiplex System (Life Technologies, Carlsbad, CA, USA). Three multiplex reactions were performed, following the design and protocols proposed in [12] with some modification (see Additional file 2). The initial PCR amplification was followed by purification with ExoSAP-IT (Amersham Biosciences, Uppsala, Sweden). A minisequencing reaction was performed and the products were purified with SAP (Amersham Biosciences). MtDNA fragment analysis was performed in SECUGEN S. L. (Madrid), and electropherograms were visualized with Peak Scanner Software v1.0 (Life Technologies). The A3796G (Hg H1b1) polymorphism was tested by Polymerase Chain Reaction-Restriction Fragment Length Polymorphism analysis (PCR-RFLP, *AciI* digestion).

It is worth noting that the level of phylogenetic resolution performed here allowed a reduction in the percentage of H* (i.e., unclassified H samples), including *i*) basal sequences with respect to the revised Cambridge Reference Sequence (rCRS, [21]), and *ii*) those mtDNA sequences that could not be ascribed to any sub-Hg because of a lack of defining markers. Proportions of H* samples of 28.8% (Huelva) and 39.3% (Granada) were observed by [18]. Here, H* frequencies in the same populations were reduced to 10.9% and 13.6%, respectively. Additional file 3 describes the molecular characterization of the 337 Hg H subjects and their assignment to specific sub-lineages.

### Statistical and phylogenetic analyses

Alignment and examination of the new mtDNA sequences, as well as calculations of statistical parameters derived from them, have been previously described [18]. Polymorphisms were scored relative to the rCRS [21]. Sub-Hg definition was determined using the coding region SNPs and the control region diagnostic positions. The nomenclature used was that of PhyloTree “mtDNA Build 16” (http://www.phylotree.org/). Mutations 16182C, 16183C and 16519 were not considered for the phylogenetic analyses.

Differences in Hg H profiles within Andalusia were tested using the *F*_*ST*_ pairwise index based on both H internal composition and sequences in ARLEQUIN 3.5 [22]. A χ^2^ test was performed to assess differences in Hg H structure between western and eastern Andalusia, and the corrected typified residuals (IBM SPSS Statistics 19) were used to determine the statistical significance between percentages.

For population comparison analyses, we constructed an updated dataset including 93 western Mediterranean and Near Eastern populations with a sample size ≥50, selected from published works (Additional file 4 **)**. The population compilation followed a number of criteria. First, we only considered those mtDNA sequences that had been tested for T7028C because assignment to Hg H is not feasible using the control region sequence alone. Second, the definition of H sub-Hgs usually requires testing the coding region (e.g., G3010A for H1, T6776C for H3). We considered control (HVS-I, range 16051-16400) and coding regions that had been tested at a resolution comparable to that used here. We initially used this dataset (which comprises 18,622 individuals of whom 6011 belong to Hg H) to describe the distribution of Hg H as a whole. We then selected only those studies (*n* = 71, underlined in Additional file 4) that dissected clade H in some phylogenetic detail (i.e., by genotyping coding variants) for the subsequent analysis of the clade internal structure. When more than a high-resolution analysis of a population was published, we pooled the samples. For northern Africa, we combined Arab and Berber populations, following previous studies in which the differences in clade H profiles in Maghrebian populations were shown to depend primarily on geography instead of culture [17].

The internal structure of Hg H in Europe and the Mediterranean region was evaluated by Hierarchical Cluster Analysis (HCA) (SPAD software, [23]). Other analyses of population structure (e.g., Analysis of Molecular Variance, AMOVA) were performed in ARLEQUIN v. 3.5 [22].

For sub-lineages observed 35 or more times, the coalescence age (time to most recent common ancestor, TMRCA) was estimated by converting the value of the *rho* (ρ) statistic into years using the corrected mutation rate for HVS-I proposed by [24]. This parameter is defined as the mean sequence divergence from the inferred ancestral haplotype of the lineage in question. For sub-clades H1 and H3, median-joining networks were obtained with the program Network 4.5 (http://www.fluxus-engineering.com) [25]. All samples used in the network analysis were screened at high phylogenetic resolution (underlined populations in Additional file 4).

Surface interpolation maps of the frequencies of Hg H and some of its main sub-clades were obtained with ArcGIS 10.1 using Ward’s linkage algorithm (see [18] for details). If available, we used the specific sampling locations; otherwise, we considered the capitals of countries or regions as the sampling locale (see black dots in Additional file 5). The existence of gradients or clinal variations for clade H as a whole, and for its major sub-Hgs (i.e., H1 and H3), were further tested by means of spatial autocorrelation analysis. The Moran’s I indices, calculated within ten distance classes, were determined using PASSaGE v.2 [26].

## Results

### Dissection of Hg H in Andalusia

The observed frequencies of Hg H and its sub-Hgs in Andalusians are shown in Table 1. The overall frequency (44%) was similar to other Iberian populations (42% on average). Among the 337 Hg H samples, 41 internal branches were observed, of which 24 had a frequency < 0.01. Hg diversity estimates (±SD) varied between 0.847 ± 0.027 in the west (Huelva) and 0.888 ± 0.013 in the east (Granada). In comparison, the Basques had the highest frequency of Hg H (∼55%) and the lowest genetic diversity (0.646 ± 0.041, estimated from data in [13]). This finding can be explained because most Basques carrying Hg H belonged either to the H1* paragroup or to some autochthonous H1 sub-branches (amounting to ~60% of mtDNA clade H variation).

**Table 1:** Frequencies of H lineages and sub-lineages in Andalusia (Southern Iberia)

Haplogroup	W-Andalusia (*Huelva*, N_T_ = 280)		E-Andalusia (*Granada*, N_T_ = 470)	
	*N*	%	*N*	%
***H***	***110***	***39.29***	***227***	***48.30***
H*	**12**	**10.91**	**31**	**13.66**
**H1**	**48**	**43.64**	**73**	**32.16**
H1*	38	34.55	58	25.55
H1a	–	–	1	0.44
H1a1	1	0.91	–	–
H1b1	–	–	5	2.20
H1c3	4	3.64	–	–
H1e1a3	–	–	5	2.20
H1k	1	0.91	–	–
H1t1a1	–	–	1	0.44
H1ah1	3	2.73	–	–
H1ba	–	–	1	0.44
H1bf1	1	0.91	2	0.88
**H2**	**3**	**2.73**	**3**	**1.32**
H2a1	3	2.73	1	0.44
H2a2b	–	–	2	0.88
**H3**	**16**	**14.55**	**41**	**18.06**
H3*	14	12.73	30	13.22
H3c	–	–	10	4.41
H3c2	2	1.82	1	0.44
**H4**	**3**	**2.73**	**12**	**5.29**
H4a1	1	0.91	12	5.29
H4a1a4b1	2	1.82	–	–
**H5**	**5**	**4.55**	**17**	**7.49**
H5*	4	3.64	6	2.64
H5a	–	–	5	2.20
H5a3a1	–	–	5	2.20
H5a4a	1	0.91	1	0.44
**H6**	**8**	**7.27**	**16**	**7.05**
H6a	6	5.45	9	3.96
H6a1a1a	–	–	4	1.76
H6a1a7	2	1.82	2	0.88
H6a1b4	–	–	1	0.44
**H7**	**5**	**4.55**	**7**	**3.08**
H7*	5	4.55	6	2.64
H7h	–	–	1	0.44
**H10**	**3**	**2.73**	–	–
H10a1	1	0.91	–	–
H10e	2	1.82	–	–
**H11**	–	–	**5**	**2.20**
H11*	–	–	4	1.76
H11a	–	–	1	0.44
**H13**	–	–	**9**	**3.96**
**H17**	**1**	**0.91**	**5**	**2.20**
H17*	1	0.91	3	1.32
H17c	–	–	2	0.88
**H18**	**5**	**4.55**	**1**	**0.44**
**H20**	–	–	**4**	**1.76**
H20*	–	–	1	0.44
H20a	–	–	3	1.32
**H27**	**1**	**0.91**	**1**	**0.44**
**H36**	–	–	**1**	**0.44**
**H82**	–	–	**1**	**0.44**

The first row on the left (in italics) indicates the frequency of the whole Hg H in each population. From there below, frequencies indicate the proportion within the Hg. Main Hg H sub-clades are highlighted in boldface. Asterisks (*) indicate those samples unclassified at the present resolution level or belonging to a specific paragroup

Sub-Hgs H1 and H3 accounted for 53% of Hg H [H1: 43.64% (Huelva) vs 32.16% (Granada); H3: 14.55% (Huelva) vs 18.06% (Granada)]. These proportions were similar to those observed in other southwestern European populations [12]. Both H1 (the typical H sub-Hg in western Europe) and H3 had the highest frequencies in the Basque-Cantabrian area of Spain. In northwestern African populations (i.e., Morocco and Algeria) H1 was found in similar proportions (~50%) to those in the Iberian Peninsula [15, 17, 27–29]. H3 varied from 8% in Moroccans to 12% in Tunisians. H3 was scarce or even absent in southern Italian, Near/Middle Eastern and Caucasian populations [9, 16].

Clade H1 was more diverse in western (0.940 ± 0.013) than eastern (0.865 ± 0.442) Andalusians. Curiously, most of the identified H1 derived branches were not shared between these two southern Iberian populations; for example, H1a1, H1c3, H1k and H1ah1 were only found in Huelva, whereas H1b1, H1e1a3, H1t1a1 and H1ba were only detected in Granada. H1 sub-lineages were generally observed at frequencies <5% of the whole Hg H group (Table 1).

When analyzing the variation of Hg H in Galicia, the most northwestern region of Spain, Álvarez-Iglesias et al. [12] recognized H1c3, H1k and H1ba as mtDNA lineages confined to the Atlantic side of Iberia. The new genetic data provided here enlarges this previously studied range to the southwestern end of the Peninsula. Behar et al. [13] defined H3c2a as a Basque-specific maternal lineage (3.30% of Hg H). H3c has been encountered in eastern Andalusia (4.41% in the present study), southern Italy [6, 30, 31] and central/eastern Europe [32, 33]. The rare lineage H1bf1 (observed in non-polymorphic frequencies among Andalusians) has been only found in Iberia [12, 14, 34]. Haak et al. [35] reported 69 new ancient samples from Early Neolithic to Late Bronze Age, which contained around 20% of Hg H lineages (see Extended Data Table 2, therein). Most of the samples came from Germany, while three were from the Yamnaya culture (Early Bronze Age, Russia) and other from La Mina (Middle Neolithic, central Spain). The most frequent ancient sampled H lineages are H1, H3 and H13, being La Mina sample H1. According to these findings, Hg H was present in Iberia at least from Middle Neolithic and some of its lineages could have been introduced there from central and eastern Europe. Nevertheless, the number of ancient mtDNA samples should be increased considerably to state, together with the most abundant contemporary ones, clearer, well-supported scenarios on the peopling of Europe.

Hgs H5 and H6 are the next major contributors within Hg H in Europe. In the present study, H5 varied between 4.5 and 7.5%, with a high proportion belonging to H5a (4.84%) (5/17 Granada Andalusians) (Table 1). Similarly, H6a was well-represented, whereas its sister branch, H6b, was not found in either of the two analyzed samples. A comparable result was previously described in northern Africa (e.g., Algeria) [27]. H6a and H6b displayed contrasting phylogeographic patterns, with the former occurring at high frequencies in Europeans, while the latter occurred more often in Near/Middle Eastern and Arabian populations (see [6, 34, 36]).

Lineages H11, H13, H20, H36 and H82 were found only in eastern Andalusians. Lineage H13 was relatively frequent across southern and eastern Iberian Mediterranean coasts [e.g., Andalusia (Granada): 4%, present study; Catalonia: 5%], and higher proportions (~10%) have been recorded in mainland Italy and Sardinia. H20 (1.80%, present study) had few representatives in Iberia [12, 17]. H36 (16070) and H82 (16220), described here by control region mutations, were observed at frequencies <1% of the whole Hg H; this finding is in close agreement with observations in other Iberian/European and Near/Middle Eastern populations [8, 9, 30, 31, 37]. Surface maps showing frequency distributions of some other specific H sub-Hgs across Europe, the Mediterranean Basin and Southwest Asia are provided in Additional files 6, 7, 8 and 9.

Hg H profiles in Andalusia exhibited substructuring (*F*_*ST*_ = 0.0065; *P*-value = 0.0180), and genetic differentiation was significant according to a χ^2^ test (χ^2^ = 30.91; d.f. = 16; *P-*value = 0.014). The analysis of the corrected typified residuals further suggested that sub-Hgs H18, H10, H1 and H13 (in descending order of significance) were the main contributors (95%, C.I.) to the observed differences. The above values closely matched those obtained with sequence data (*F*_*ST*_ = 0.007; *P-*value = 0.027). When combining control region sequences and coding region polymorphisms, we found 158 different haplotypes among the 337 H individuals (46.9%), with only a small percentage of them (17/158;11%) shared between western and eastern Andalusians.

**Fig. 2:**
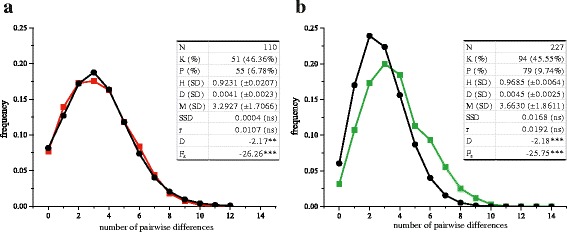
Mismatch distributions for Huelva (**a**) and Granada (**b**) and statistical parameters of control region sequences. For both cases, expected distributions are shown in *black*. K: number of different sequences and percentage of sample size in parentheses; P: number of polymorphic sites; H: haplotype diversity and standard deviation; D: nucleotide diversity; M: mean number of pairwise differences; SSD: sum of squared deviations between the observed and expected mismatch distribution; r: Harpending’s raggedness index; D and Fs are, respectively, the Tajima’s and Fu’s tests of selective neutrality. The last 4 indices are presented with their significance values, as ns (non-significant), **P < 0.01; ***P < 0.001

Figure 2 displays the mismatch distributions among Hg H sequences. Scenarios of recent population expansion for Hg H in Andalusia are supported by the significantly negative values of Tajima’s (*D*) and Fu’s (*Fs*) neutrality tests. The non-significant sum of squared deviations (SSD) between observed and expected distributions of pairwise differences, along with the Harpending’s index (*r*) value also support the above statement. The entire distribution of observed Hg H control region haplotypes (*n* = 337) had a high goodness of fit (R^2^ = 0.879), which serves as a quality indicator reflecting the lack of sampling bias. Haplotype diversities [0.923 ± 0.021 (Huelva) and 0.969 ± 0.006 (Granada)] were higher than those found in the northern extremes of the Peninsula (e.g., Galicia: 0.800 ± 0.038; Cantabria: 0.875 ± 0.042, [12]). In North Africans, haplotype diversity was between 0.860 ± 0.060 in Moroccans and 0.970 ± 0.010 in Tunisians. The latter figure seems to be explained by a strong influence from the Near East [17]. In consequence, autochthonous Andalusians from Granada were distinguished by a high prevalence of clade H with internal diversity, reflecting interesting influences from other Mediterranean populations.

### Phylogeography of Hg H on both sides of the Strait of Gibraltar

**Fig. 3:**
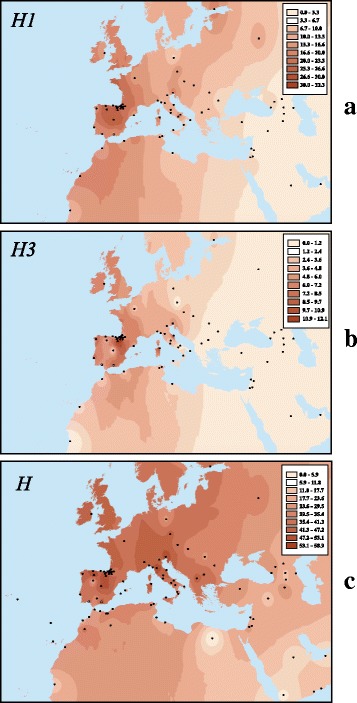
**a**, **b**, **c**. Interpolated frequency surfaces of clade H and its main sub-clades (H1 and H3). Frequencies (%) are showed in a colour scale. See information about the populations used in Additional files 4 and 5. Map templates were taken from Natural Earth free map repository (http://www.naturalearthdata.com/)

Figure 3 shows surface maps based on frequency distributions of clades H, H1 and H3 in Europe and surrounding areas. The maps make more than apparent the presence of spatial structure, with frequencies decreasing from west to east across both the northern and southern sides of the Mediterranean (Moran’s correlograms Bonferroni corrected significance of 0.0000). A pattern characterized by positive (and significant) autocorrelation values for short distances and significant negative autocorrelations at long distances (further details in [38]) emerged for H1 when considering only the European population points; the correlogram exhibited a linear gradient (Additional file 10 B) with no inflection points.

In connection with the above findings, major differences for Hg H composition were also detected between populations located in the west and east of the Mediterranean Basin (*F*_*CT*_ = 0.065, *P*-value <0.001), with H1 contributing more prominently to that structure. Roostalu et al. [9] interpreted these distinctive profiles as the result of a limited maternal gene flow after the Last Glacial Maximum (LGM) between the geographic extremes of the Mediterranean.

**Fig. 4:**
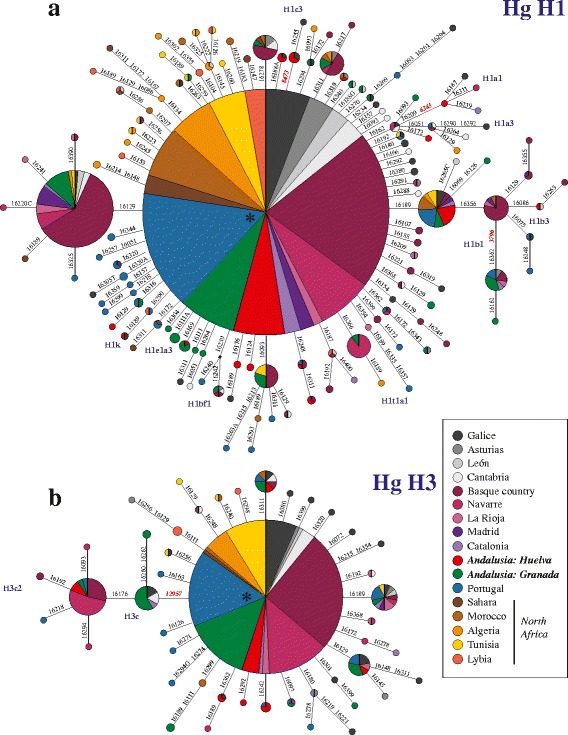
Median-joining networks for sub-Hgs H1 (**a**) and H3 (**b**) in Iberia and Northern Africa. HVS-I sequences (n_H1_ = 708; n_H3_ = 264) are shown along with some coding-region positions (*in bold red*). All positions are scored against the rCRS. Sub-Hgs are filled in *blue*. Transversions are specified with the base change after the mutation. The *asterisks* show the basal nodes (nps. 3010 for H1; nps. 6776 for H3). Circle sizes are proportional to the haplotype frequency. See Additional file 4 for details on populations used

Figure 4 shows the networks of 708 (a) and 264 (b) mtDNA sequences/haplotypes found in 12 Iberian and 5 North African populations for sub-Hgs H1 and H3, respectively. Both networks are strongly star-shaped. Interestingly, the H1 basal node (Fig. 4a) contains the entire spectrum of population samples used in the analysis, representing 46.5% of the total H1 sequences. The same is true for H3 (56.8% of the total H3 sequences). Maternal relationships between Iberia and North Africa rely on a concomitant nodal presence of sequences from those regions in most of the sub-Hgs, with Iberians being the main contributors to those nodes (e.g. H1b initial radiations, Fig. 4a). Most tip branches stemming directly from basal nodes (see the multiple radiations around them defined by single mutations) were unique and belonged in many cases to individuals of North African origin. In Hg H1 in particular, branches indicate a distinct radiation in North Africa compared to Iberia (see upper-left corner in Fig. 4a).

Table 2 shows the number of different H1 and H3 haplotypes encountered in Iberia and Andalusia vs the Maghreb and Morocco, along with the proportions of haplotypes shared among these regions and populations. Interestingly, the observed proportion of haplotypes shared between Andalusians and Moroccans, relative to the total observed haplotypes in the region with lower abundance, was 83% (5/6) for H1 and 50% for H3 (2/4). The proportions were lower [H1: 35% (7/20) and H3: 33% (2/6)] when Andalusia was compared to the rest of Maghreb. When analyses were based on other Iberian vs Morocco and other Iberian vs other Maghrebian populations, percentages ranged between 50 and 100%.

**Table 2:** Cases (above diagonal) and percentages (below diagonal) of different and shared mtDNA haplotypes (non-singletons) for H1 and H3 Hgs between Andalusia, Iberia^a^, Morocco and Maghreb^b^

Hg H1	Andalusia *(n = 26)*	Iberia^a^ *(n = 51)*	Morocco *(n = 6)*	Maghreb^b^ *(n = 20)*
Andalusia	–	22	5	7
Iberia^a^	84.62	–	6	13
Morocco	83.33	100.00	–	4
Maghreb^b^	35.00	65.00	66.67	–
Hg H3	Andalusia *(n = 10)*	Iberia^a^ *(n = 14)*	Morocco *(n = 4)*	Maghreb^b^ *(n = 6)*
Andalusia	–	8	2	2
Iberia^a^	80.00	–	2	4
Morocco	50.00	50.00	–	2
Maghreb^b^	33.33	66.67	50.00	–

without ^a^Andalusia and ^b^Morocco

The coalescence ages of some H sub-Hgs based on the HVS-I region are displayed in Table 3. With the exception of H2, H11 and H20, which exhibit the oldest ages [23,648 ya (Hg H2); 21,200 ya (H11); 17,789 ya (Hg H20)], the remaining H sub-Hgs were compatible with either a last postglacial or early Holocenic origin. Age values for H1 and H3, although with overlapping confidence intervals, have deeper point estimates in Iberia than in North Africa (H1: 13,400 ya in Iberia vs 10,500 ya in North Africa; H3: 9500 ya in Iberia vs 8100 ya in North Africa). These findings could be explained by an old introgression from Iberia, represented by the Andalusia case study, to neighboring North Africa, probably as a consequence of the same population movements that caused resettlement within Europe [15].

**Table 3:** Coalescence age estimates for the main H-subclades

Sub-clade	*N*	*ρ* (HVS-I)	σ	Age (years)	95% C.I.
H1	1524	0.99	0.21	16,480	[9687–23,273]
H1 Iberia	591	0.80	0.20	13,375	[6892–19,859]
H1 North Africa	191	0.63	0.11	10,478	[6756–14,200]
H2	244	1.42	0.70	23,648	[899–46,397]
H3	391	0.78	0.20	13,085	[6442–19,728]
H3 Iberia	236	0.57	0.15	9469	[4420–14,518]
H3 North Africa	37	0.49	0.17	8113	[2526–13,700]
H4	129	0.57	0.13	9437	[5243–13,632]
H5	324	0.61	0.14	10,140	[5686–14,594]
H6	177	0.78	0.19	13,002	[6931–19,074]
H7	120	0.66	0.19	10,979	[4756–17,202]
H10	69	0.88	0.41	14,743	[1286–28,201]
H11	59	1.27	0.41	21,200	[7823–34,577]
H13	120	1.00	0.20	16,677	[10,154–23,200]
H20	46	1.07	0.50	17,789	[1353–34,225]

Values of the parameter ρ were based on HVS-I sequences detailed in Additional file 4 and transformed into years by using the corrected molecular clock proposed by [24]

**Fig. 5:**
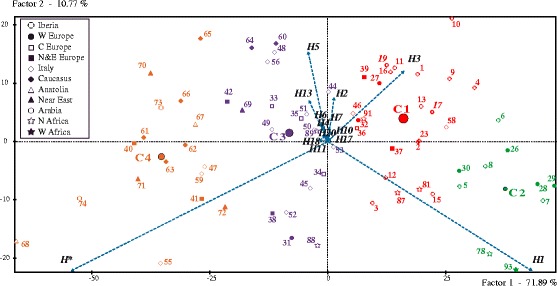
Hierarchical Cluster Analysis (HCA) of 71 populations based on their Hg H sub-structure. Only high-resolution studies were considered for this analysis (see populations underlined in Additional file 4). The sub-clades used here are depicted as vectors. Affiliation to each of 4 clusters discussed in the text is colour-coded. Each geographic affiliation has a characteristic symbol (see legend). Populations are coded as in Additional file 4. Our Andalusian populations correspond to numbers 17 (Huelva) and 19 (Granada)

The multivariate HCA depicted in Fig. 5 displays patterns of Hg H frequency variations among 71 selected population samples from Europe, the Mediterranean Basin, and other surrounding regions (dataset in Additional file 4). Factor 1 explained 71.89% of the total variance, whereas Factor 2 explained only 10.77%. The HCA identified four population clusters and the Hgs H*, H1, H3, H5 and H13 (see vectors in the plot) significantly determined a population’s position. When the inertia decomposition on the first two axes was computed, the inertia quotient after consolidation was 0.812. This value was coherent with the number of major branches shown by the tree and confirms that a high proportion of the data variation was explained by these four groupings.

Cluster 1 (C1) was the most numerous (*n* = 24) and was largely composed of Iberian samples [*n* = 14 out of 18; the two Andalusian samples here reported, shown in *italics,* are part of this cluster], together with others from the rest of Europe and North Africa (e.g., Morocco, Algeria and Libya). C1 was characterized by sub-Hgs H1 (cluster mean = 41.34%, *P-*value = 0.006) and H3 (mean = 15.20%, *P-*value = 0.000). C2 (*n* = 10) could be described as the “Basque” cluster, with H1 showing strong statistical significance (mean 64.22; *P*-value = 0.000) in defining the Basque mtDNA gene pool. C3 was primarily characterized by Hg H5 (mean = 9.5%, *P-*value = 0.016) and comprised a high number of populations (*n* = 20) with a major Italian component along with samples from Tunisia (#88, #89 in Additional file 4 dataset), the Near/Middle-East and the Caucasus. Finally, cluster C4 (*n* = 17) was significantly influenced by H* (mean = 58.25%, *P-*value = 0.000) and more weakly by H13 and H20. The mosaic of populations that made up this grouping originated primarily in the Middle East, the Caucasus and southwestern Asia (*n* = 12), with a few others (*n* = 5) from Italy and central Europe. The H10, H3 and H1 lineages were particularly underrepresented in C4.

Datasets from Iberia and North Africa used in the HCA were also considered for further assessments of population structure. There was a lack of significant genetic differences between northern Spanish population groups (i.e., Galicians, Asturians, Cantabrians and Basques) and those from the southern end of the Peninsula (Andalusians, present study) (see Table 4). Other comparisons of Andalusia vs Maghreb or Andalusia vs Morocco/Algeria yielded similar results, i.e., the absence of population structure. These findings were unsurprising given the demonstrated genetic affinities for maternal heritage, especially among populations from Iberia and northwestern Africa around the Strait of Gibraltar. These close genetic relationships were easily observed in the HCA (Fig. 5)**,** where Morocco (#85) and Algeria (#87) (for details see Additional file 4) grouped together with most Iberians in the C1 cluster.

**Table 4:** *F*_*SC*_ and *F*_*CT*_ indices obtained by AMOVA for several groups of northern and southern Mediterranean populations

	Fixation Indices			
Population Groups	*F*_*SC*_	% total variance	*F*_*CT*_	% total variance
Southern Iberia (Andalusia) (2)^a^ vs Northern Iberia (12)^b^	0.01921***	1.90	0.01022 (n.s.)^e^	1.02
Southern Iberia (Andalusia) (2) vs Spanish Basque Area (8)^c^	0.01944***	1.90	0.02117 (n.s.)	2.12
Iberian Peninsula (18) vs Maghreb (6)^d^	0.02035***	2.01	0.01034 (n.s.)	1.03
Iberian Peninsula (18) vs Morocco, Algeria (2)	0.01838***	1.83	0.00592 (n.s.)	0.59
Iberian Peninsula (18) vs Italy (14)	0.02284***	2.17	0.05207***	5.21
Andalusia (2) vs Maghreb (6)	0.01590***	1.57	0.01017 (n.s.)	1.02
Andalusia (2) vs Morocco, Algeria (2)	0.00224 (n.s.)	0.22	0.02106 (n.s.)	2.11

Results are based on mtDNA clade H composition

All values have been corrected for multiple testing using Bonferroni

^a^Number of populations per group

^b^Including from west to east the Spanish northern regions of Galicia, Asturias, Cantabria and Basque Country

^c^Including the Basque Country and the neighboring territory of Navarra

^d^Including Morocco, Algeria, Tunisia and Libya

^e^n.s. not significant; ***P < 0.001

## Discussion

The Iberian Peninsula, given its geographic position and role as a human refuge during the last glaciation, is an especially appropriate territory to study bidirectional migrations from Europe to North Africa and back following the LGM. The analysis of continent-specific mtDNA Hgs, such as H in Europe, the high frequencies of some H sub-Hgs in the western extreme of the Mediterranean and the estimated coalescence dates, mostly concentrated ~10,000 ya, make mtDNA Hg H a suitable candidate to investigate these migrations. Our study on Hg H with high phylogenetic resolution in southern Iberia highlights how intertwined and strongly rooted the evolutionary history among western Mediterranean regions has been, based on maternal heritage. Signals of ancient migrations across the Strait of Gibraltar and its surrounding maritime area have also been observed using other genomic markers [39].

Today, there is empirical evidence that the contribution of European and African mtDNA lineages is not equivalent on the opposite coasts of the western Mediterranean. The traces left by African mtDNA clades U6, M1 and L in the European gene pool are greatest in the Iberian Peninsula, with the highest frequencies of occurrence (~15%) in western Andalusia [18]. In contrast, frequencies of Hg H in northwestern Africa were comparable to those found in southwestern Europe (e.g., Iberian Peninsula, 40-45%). The described scenario suggest that the Iberian Peninsula served as both a sink (*recipient of genes*) and source (*donor of genes*) population in relationship to Africa. The role of Iberia in the spread of genes from Africa to the rest of Europe would also be paralleled by the transmission of other genetic traces southward along the Mediterranean coast and across the Strait of Gibraltar. The lower demographic size in northwestern Africa in relation to Iberia during past millennia would have favored processes increasing the frequency of Hg H in the former region.

As the climate warmed after the end of the LGM, wide ranging movements of populations through Europe became possible [40]. Those post-LGM human movements from the three major refugia in the Mediterranean, Iberia, Italy and the Balkans, also reached the North African fringe [41]. In a broader context, the genetic structuring of Europe revealed by genome-wide (GW) studies primarily exhibits a latitudinal trend [42, 43], which coincides well with post-glacial processes and other later demographic events. The general GW autosomal pattern is further enriched by interesting regional variations. For example, the Italian and Iberian peninsulas share little recent common ancestry with other European populations, a fact that has been linked to old substructuring [44]. Our Iberian mtDNA data [18, 19] point to another possible interpretation for this local specificity: the genetic influence from the neighboring African continent.

Phylogeographic and phylogenetic analyses performed in the present study suggest that the main H sub-Hg indicators of post-glacial population expansions from the Iberian refuge were H1 and H3, the most frequent maternal lineages in Iberia. The time depths calculated for these sub-clades in Iberia and the Maghreb, together with the presence of specific H variants in some North African populations –revealed at a deep phylogenetic resolution (e.g. H1v1, H1w, and others in Tuareg from Libya [16])– it would support scenarios of ancient radiations in the direction Iberia-to-North Africa. Accordingly, the genetic structure analysis of Mediterranean populations by AMOVA demonstrated that Italy was significantly different from Iberia (see Table 4) and that this dissimilarity was not as visible between Iberian and Maghrebian populations. These findings suggest that gene flow between Europe and northwestern Africa, involving Hg H, would have occurred primarily through Iberia. The HCA also provided strong support for this assertion.

Interestingly, the patterns of variation displayed by Hgs H1, H3 and H5 (see surface maps in Fig. 3 and Additional file 6), together with other evidence emerging from the HCA, suggest more than one post-glacial migration from the Franco-Cantabrian refuge. In a first, more geographically restricted migration, Basque populations with high levels of H1 would have been the primary participants. A second migration would have harbored the most recent sub-Hg H3 and reached the Maghreb. A probable third migration, with a center of origin in the eastern Mediterranean, would have carried the H5 mtDNA lineage into Italy and, to a lesser extent, the western Mediterranean, with a reduced impact in northern and western Iberia. These human movements could have been conducted, at least in part, by sea. It is interesting to note that the native population from Sardinia exhibits high frequencies of H1 and H3, a fact that would indicate early maritime relationships with the near mainland Europe.

An interesting result of the H1 and H3 star-like networks of the Iberian and Maghrebian populations (top left in Fig. 4a) was the number of haplotypes found two or more times in North Africa. This may represent an incipient molecular radiation, indicative of early human migrations between the western Mediterranean shores. Other derived variants from larger nodes occurred at variable frequencies (e.g., the most predominant in the H1 skeleton network occurs 74 times) and African individuals contributing to these nodes were scarce. This suggests that historical migratory flow between Iberia and the Maghreb had a lower genetic influence than ancient human movements. As shown in Table 2, the proportion of shared haplotypes between Andalusia and Morocco was high, but were considerably lower when comparing Andalusia against the Maghreb. Thus, migrations would have shown an expansion within a reduced geographic range.

The necessity of crossing a body of water to reach the African continent allows us to analyze the opportunities for navigation in early times. Broodbank [45, 46] considered any possible maritime activity in the Mediterranean before *c*. 12,000 ya to be episodic and of limited evolutionary significance. However, the presence of Mesolithic seafaring is now quite firmly established based on recent finds from Crete, the Aegean, Sardinia and Corsica. The unambiguous evidence for human presence on Mediterranean islands still dates to no more than *c*. 16,000 ya [47]. Thus, maritime contact between the two continents could have begun before 10,000 ya, the time depth for which the most frequent H Hgs can be used to detect migrations. This conclusion is strongly supported by similarities between lithic industries dominated by microliths and backed bladelets of the Iberomaurusian and Magdalenian and by Taforalt harpoon (northeastern Morocco), which has three short barbs on one edge and is contemporaneous with the Final Magdalenian in Mediterranean Spain [48]. Harpoons detected in prehistoric excavations from southern Catalonia to Malaga Bay (the Spanish Mediterranean corridor) are also characterized by having a single row of teeth. Barbed harpoons were the most common implement in the Upper Magdalenian [49] and could have been introduced to North Africa by the advancing ice. Archaeological dates confirm the simultaneity of the Epipalaeolithic-Neolithic transition in southern Spain and northern Morocco.

The rapid dispersal of innovations suggests that they were circulated through already existing networks [50]. The earliest presence of Neolithic industries in southern Iberia were dated at least 7500 calibrated ya [51]. All major elements of the Neolithic package arrived in southeastern Spain from the central Mediterranean and reached North Africa through west Mediterranean networks. However, some elements, such as pointed-based vessels, Almagra decoration, and lentils, were subsequently modified in North Africa before being dispersed to Iberia [52].

The first maritime contact would have been associated with fishing in waters more distant from the coast and with increasingly larger watercrafts used for the capture of large fish. In Nerja cave (Malaga), the remains of large sea mammals (e.g., monk seal, harbor seal, dolphin and large cetaceans of the *Delphinidae* family) have been found [49]. The route stopping on the small island of Alboran, between eastern Morocco and southeastern Andalusia, is particularly interesting for crossing the Alboran Sea, with land permanently in sight [19]. A bidirectional movement of women could occur as part of these dynamic journeys. The transport of the Neolithic package, including farmers, domesticated animals, seeds and tools, must have required sea crafts of considerable size, and this could increase the intensity of potential admixture. Given the high frequencies of Hg H on both shores, this gene flow would have been intense for thousands of years.

Reed rafts tied with green stems were the probable primitive watercrafts used in the Alboran Sea. The use of leather sails is also possible, since leather treatment technology is ancient. The Libyan Desert, which is adjacent to the coast, would have been a stronger barrier to human movement than maritime travel. Therefore, prehistoric bidirectional seafaring in the westernmost Mediterranean seems quite well evidenced by the archaeological record and it is also supported by genetic data. Protohistoric and historic episodes reinforced the connections between the Maghreb and Iberia across the administrative and politic unities established during the Roman Empire and, later, with the Muslim expansion, with relevant sociocultural and economic consequences. Thus, the westernmost extreme of the Mediterranean likely did not represent a true physical barrier to gene flow between both continents.

The patterns of variation in the Y-chromosome between western and eastern Andalusians, based on 416 males, have also been investigated for a set of Y-Short Tandem Repeats (Y-STRs) and Y-SNPs [53–55], Calderón et al., unpublished data] in combination to mtDNA analyses ([18, 19] and present study). In general, for both uniparental makers, Andalusians exhibit a typical western European genetic background, with peak frequencies of mtDNA Hg H and Y-chromosome Hg R1b1b2-M269 (45% and 60%, respectively). Interestingly, our results have further revealed that the influence of African female input is far more significant when compared to male influence in contemporary Andalusians. The lack of correspondence between the maternal and paternal genetic profiles of human populations reflects intrinsic differences in migratory behavior related to sex-biased processes and admixture, as well as differences in male and female effective population sizes related to the variance in reproductive success affected, for example, by polygyny [56, 57].

## Conclusion

Here we present arguments that the western Mediterranean has not been a barrier to human gene flow and, more specifically, that the Strait of Gibraltar and adjacent areas acted as an active bridge between Africa and Europe. A pertinent example is found in Andalusia and its autochthonous contemporaneous populations, whose genetic composition is notably influenced both by the close proximity to North Africa and by intense involvement in the history of the Mediterranean.

## Additional files

Additional file 1: TaqMan design for genotyping H and H1 lineages. (XLSX 10 kb)

Additional file 2: Amplification primers and probes for the three SNaPshot multiplex reactions. (XLSX 62 kb)

Additional file 3: Mitochondrial molecular characterization of 337 Andalusians belonging to haplogroup H. Control region information is showed jointly with coding SNPs. (XLSX 65 kb)

Additional file 4: Literature compilation for haplogroup H comparative analyses. Geographic affiliations are as follows: WME (Western Mediterranean Europe), WEU (Western Europe), CEU (Central Europe), NEU (North Eastern Europe), EEU (Eastern Europe), CME (Central Mediterranean Europe), CAU (Caucasus), ANA (Anatolia), NES (Near East), ARA (Arabian Peninsula), NAF (North Africa), WAF (Western Africa). Those studies that reached a deep level of resolution inside clade H (towards the definition of specific sub-clades by coding SNP testing) are underlined. The table shows the total sample size (N) and the number of individuals classified as belonging to haplogroup H (N_H_) for each population. (XLSX 16 kb)

Additional file 5: Geographic location of the 93 populations used for comparative purposes and haplogroup frequency maps. A. Europe and the Mediterranean space. B. Detailed view of the Iberian Peninsula. See codes and references in Additional file 4. Map templates were taken from Natural Earth free map repository (http://www.naturalearthdata.com/). (PDF 722 kb)

Additional file 6: Interpolation frequency maps (% of the population) of broadly distributed H sub-clades (H2, H4, H5 and H6). Map templates were taken from Natural Earth free map repository (http://www.naturalearthdata.com/). (PDF 2058 kb)

Additional file 7: Interpolation frequency maps (% of the population) of sub-clades with a western European/Mediterranean preferential distribution (H17 and H18). Map templates were taken from Natural Earth free map repository (http://www.naturalearthdata.com/). (PDF 791 kb)

Additional file 8: Interpolation frequency maps (% of the population) of sub-clades with a central European preferential distribution (H7, H10 and H11). Map templates were taken from Natural Earth free map repository (http://www.naturalearthdata.com/). (PDF 1261 kb)

Additional file 9: Interpolation frequency maps (%) of sub-clades with an eastern European/Mediterranean preferential distribution (H13 and H20). Map templates were taken from Natural Earth free map repository (http://www.naturalearthdata.com/). (PDF 889 kb)

Additional file 10: Spatial Autocorrelation Analyses Correlograms of clade H and sub-clades with significant global values of Moran’s I after Bonferroni correction. Significant points are indicated as red circles (*P*-value = 0.05) and non-significant as white circles for 10 distance classes. Distances are shown in kilometres. The analysis was performed for the whole database (see Additional file 4), for Mediterranean populations (populations 1-23, 26-31, 41-59, 67-72, 78-92), and for Europe (populations 1-59). (PDF 425 kb)

## Abbreviations

AMOVA: Analysis of Molecular Variance
GW: Genome-Wide
HCA: Hierarchical Cluster Analysis
Hg: Haplogroup
HVS: Hypervariable Region
LGM: Last Glacial Maximum
mtDNA: Mitochondrial DNA
PCR-RFLP: Polymerase Chain Reaction-Restriction Fragment Length Polymorphism
rCRS: Revised Cambridge Reference Sequence
SNPs: Single Nucleotide Polymorphisms
STRs: Short Tandem Repeats
TMRCA: Time to Most Recent Common Ancestor
ya: years ago
