# Supplementary figures and images for: The distribution of mitochondrial DNA haplogroup H in southern Iberia indicates ancient human genetic exchanges along the western edge of the Mediterranean
Source: BMC Genet. 2017 May 19;18:46. doi: 10.1186/s12863-017-0514-6 (PMC5437654; doi:10.1186/s12863-017-0514-6)

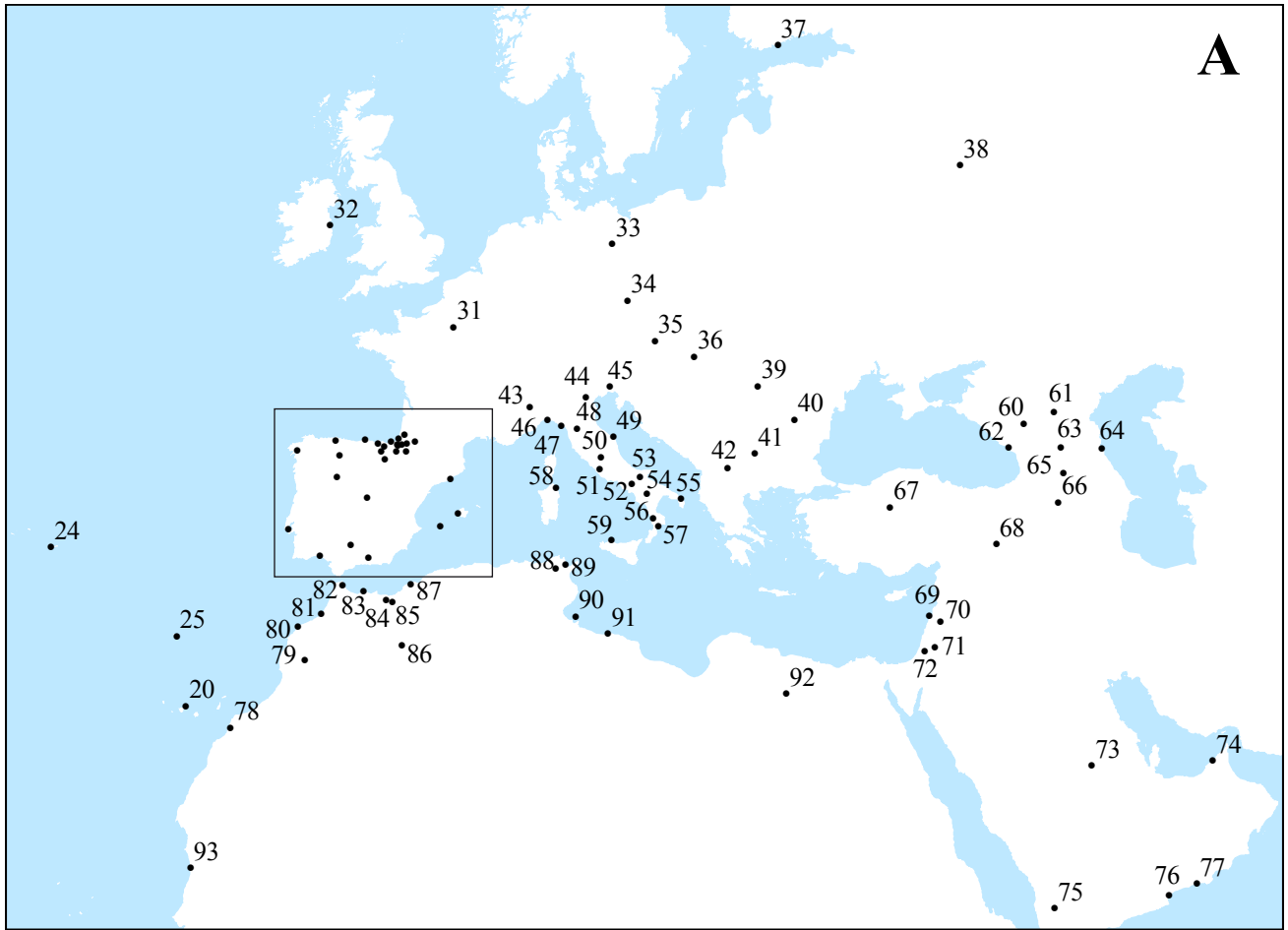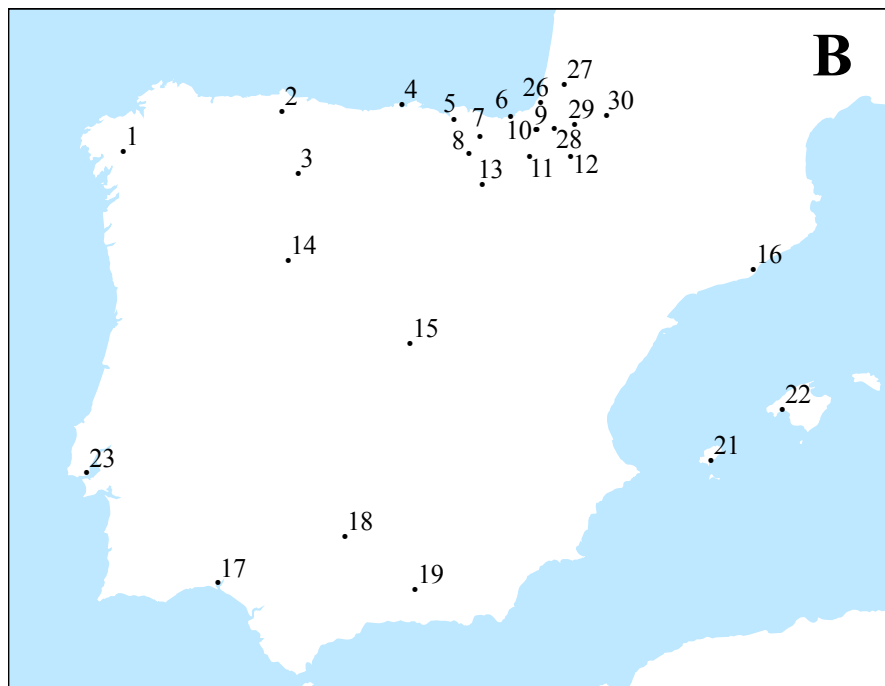

Supplement: Supplementary file 5 — Geographic location of the 93 populations used for comparative purposes and haplogroup frequency maps. A. Europe and the Mediterranean space. B. Detailed view of the Iberian Peninsula. See codes and references in Additional file 4. Map templates were taken from Natural Earth free map repository (http://www.naturalearthdata.com/). (PDF 722 kb) [file 12863_2017_514_MOESM5_ESM.pdf]

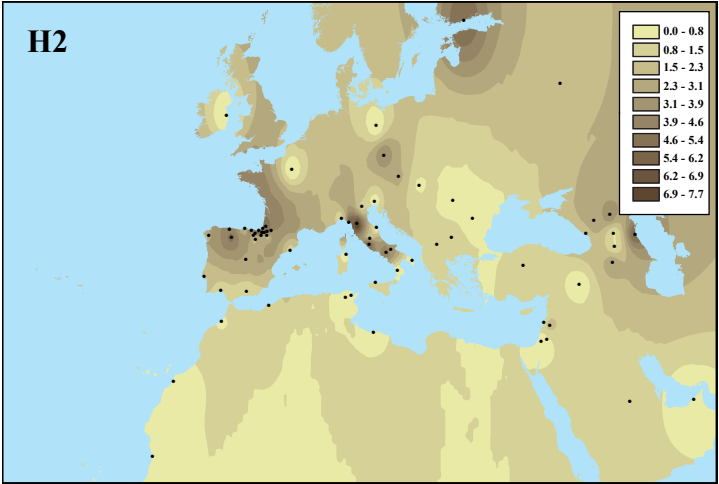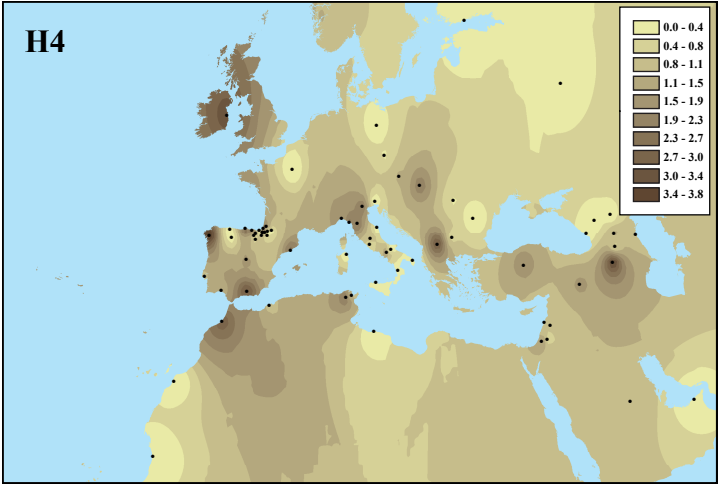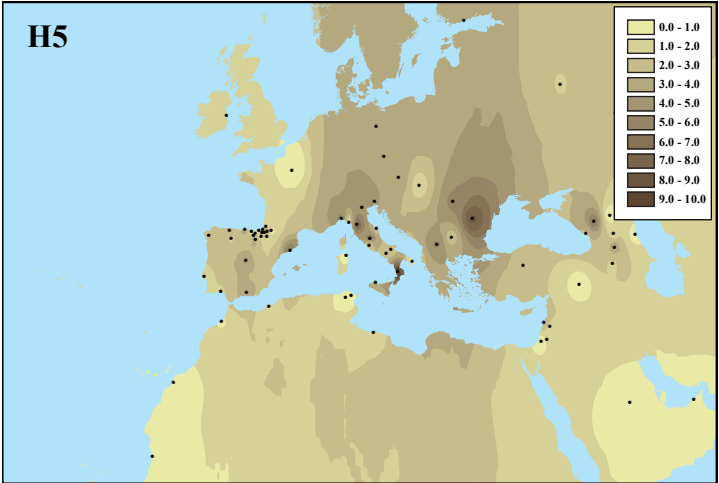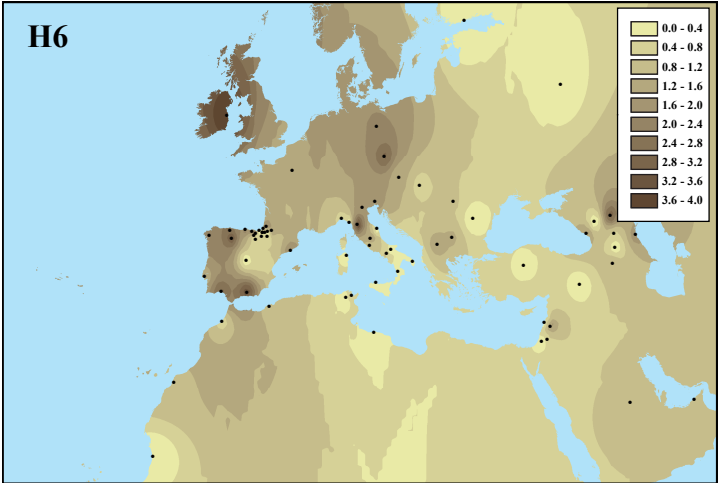

Supplement: Supplementary file 6 — Interpolation frequency maps (% of the population) of broadly distributed H sub-clades (H2, H4, H5 and H6). Map templates were taken from Natural Earth free map repository (http://www.naturalearthdata.com/). (PDF 2058 kb) [file 12863_2017_514_MOESM6_ESM.pdf]

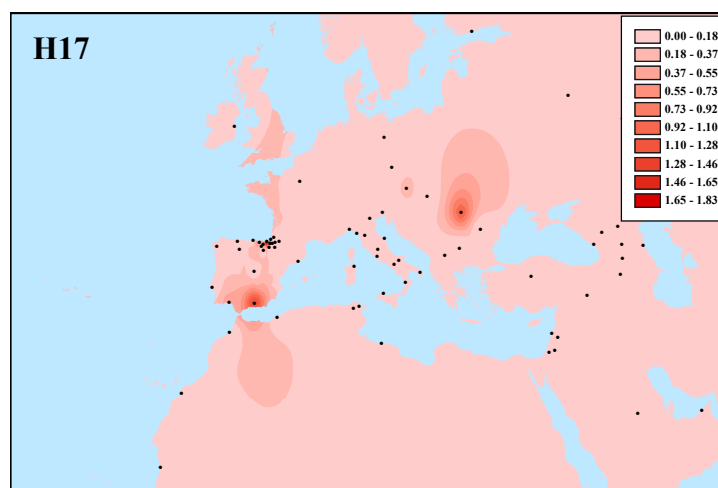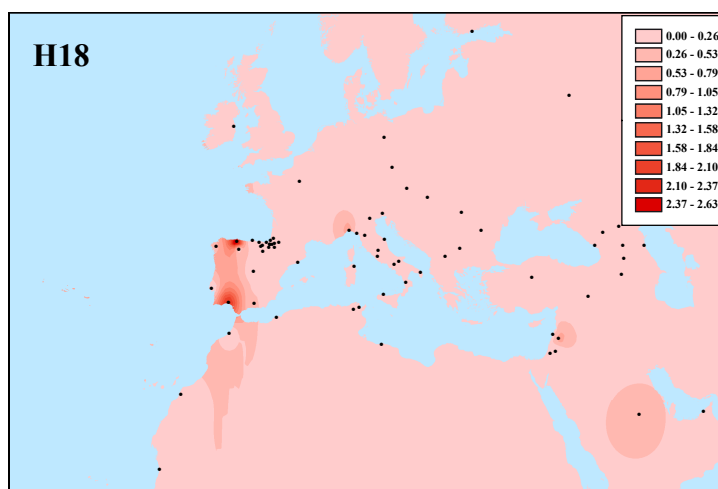

Supplement: Supplementary file 7 — Interpolation frequency maps (% of the population) of sub-clades with a western European/Mediterranean preferential distribution (H17 and H18). Map templates were taken from Natural Earth free map repository (http://www.naturalearthdata.com/). (PDF 791 kb) [file 12863_2017_514_MOESM7_ESM.pdf]

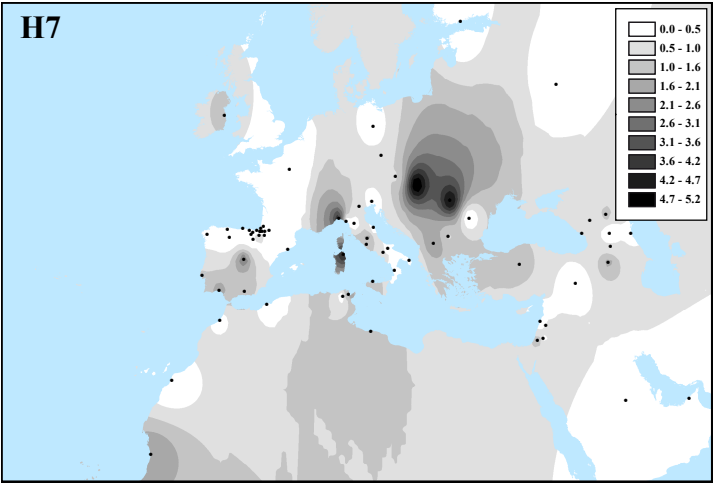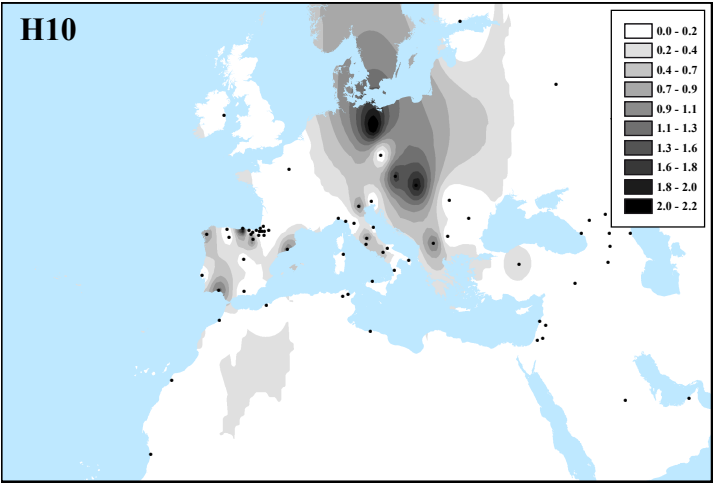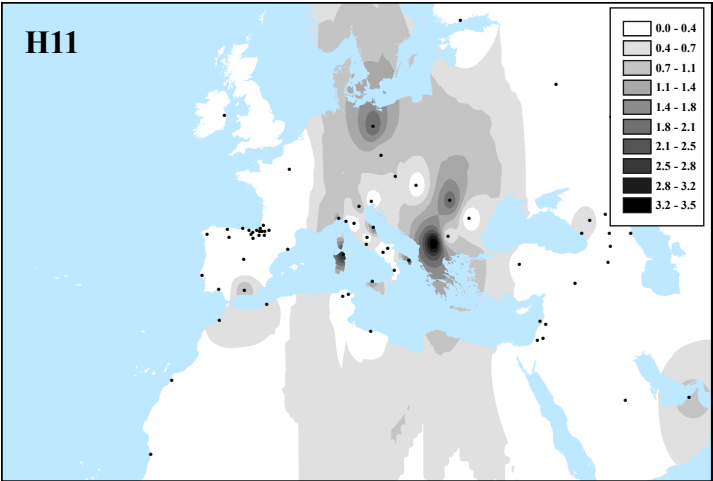

Supplement: Supplementary file 8 — Interpolation frequency maps (% of the population) of sub-clades with a central European preferential distribution (H7, H10 and H11). Map templates were taken from Natural Earth free map repository (http://www.naturalearthdata.com/). (PDF 1261 kb) [file 12863_2017_514_MOESM8_ESM.pdf]

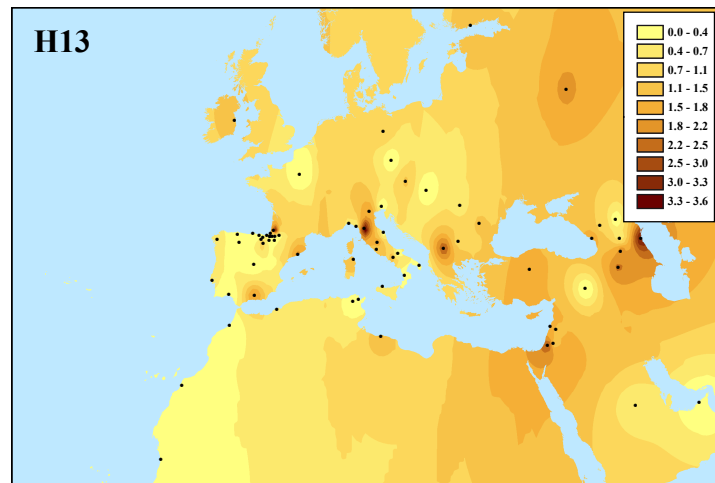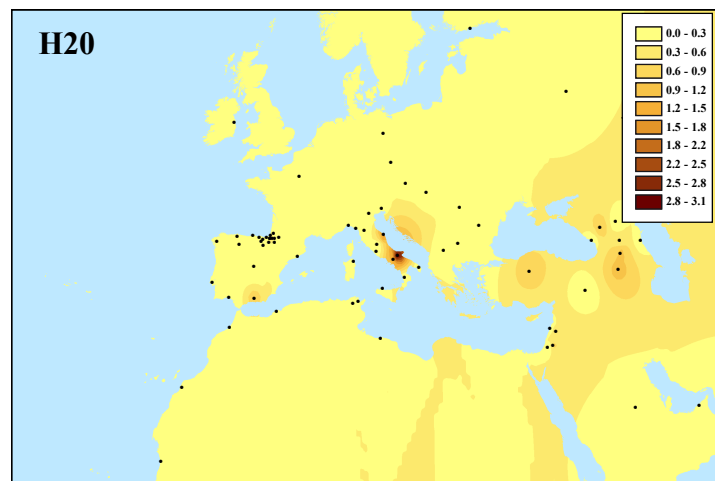

Supplement: Supplementary file 9 — Interpolation frequency maps (%) of sub-clades with an eastern European/Mediterranean preferential distribution (H13 and H20). Map templates were taken from Natural Earth free map repository (http://www.naturalearthdata.com/). (PDF 889 kb) [file 12863_2017_514_MOESM9_ESM.pdf]

A

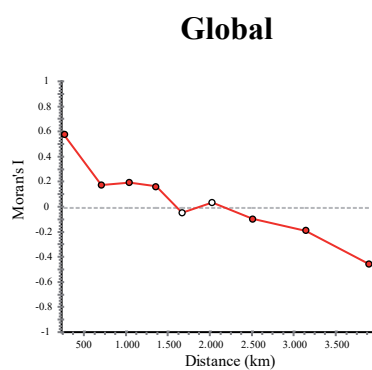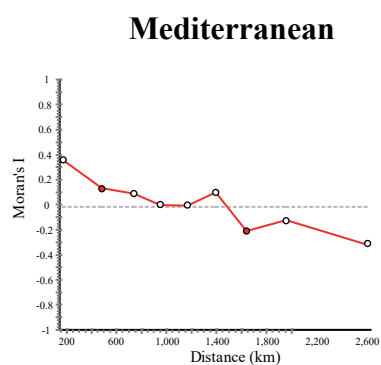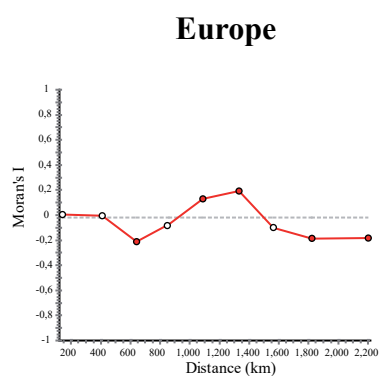

Hg H

B

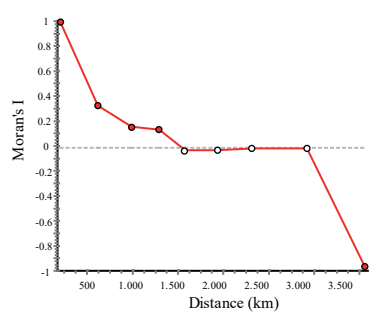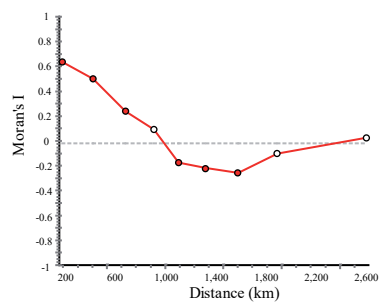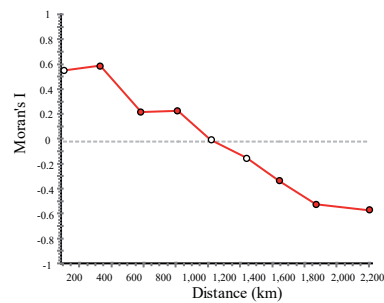

Hg H1

C

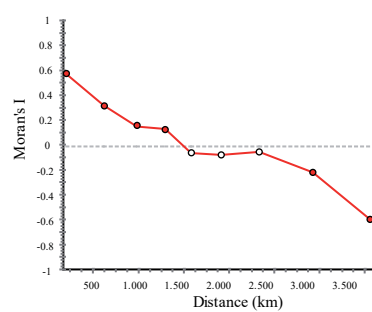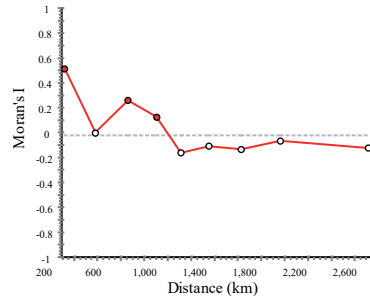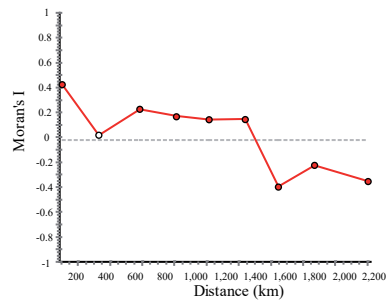

Hg H3

Supplement: Supplementary file 10 — Spatial Autocorrelation Analyses Correlograms of clade H and sub-clades with significant global values of Moran’s I after Bonferroni correction. Significant points are indicated as red circles (P-value = 0.05) and non-significant as white circles for 10 distance classes. Distances are shown in kilometres. The analysis was performed for the whole database (see Additional file 4), for Mediterranean populations (populations 1-23, 26-31, 41-59, 67-72, 78-92), and for Europe (populations 1-59). (PDF 425 kb) [file 12863_2017_514_MOESM10_ESM.pdf]
